# Will coronal alignment postoperatively be deteriorating in adult spinal deformity after long-fusion surgery?

**DOI:** 10.1186/s40001-024-01798-4

**Published:** 2024-03-25

**Authors:** Zifang Zhang, Tianhao Wang, Nianhu Li, Guoquan Zheng, Chunyang Meng

**Affiliations:** 1 Affiliated Hospital of Jining Medical University, Jining Medical University, Guhuai Road 89, Jining, 272007 China; 2 Shandong University of Traditional Chinese Medicine, Jinan, China; 3 The Spine Surgery, The Fourth Medical Center of the Chinese PLA General Hospital, Fuxing Road 28, Beijing, 100853 China; 4 Department of Orthopedic Surgery, Affiliated Hospital of Jining Medical University, Guhuai Road 89, Jining, 272007 China; 5 Department of Spine Surgery, Affiliated Hospital of Jining Medical University, Jining Medical University, Guhuai Road 89, Jining, 272007 China; 6 Department of Orthopedic Surgery, The fourth Medical Center of the Chinese PLA General Hospital, Fuxing Road 28, Beijing, 100853 China

**Keywords:** Adult spinal deformity, Coronal malalignment, Coronal balance distance, Coronal alignment, Long-fusion surgery

## Abstract

**Background:**

To investigate whether the coronal alignment (CA) will deteriorate, and identify the risk factors for coronal malalignment (CM) developing in adult spinal deformity (ASD) after long-fusion surgery.

**Methods:**

A multi-center retrospective study was performed, which included a total of 161 ASD patients who had undergone the surgical procedure of long-fusion (≥ 5 vertebras) with instrumentations in three medical centers. All of the participants were retrospectively reviewed, and subsequently assigned into the consistency group (C7 plumb line (C7PL) shifting towards the convex side of the main curve), and the opposition group (C7PL shifting towards the concave side). CM was considered if the coronal balance distance (CBD) being over 30 mm. A Kaplan–Meier curve and log-rank test were used to analyze the differences in CM-free survival during follow-up. Multivariate analysis via a Cox proportional hazards test was used to analyze the risk factors.

**Results:**

Patients showing CM equaled 35 (21.7%) at the pre-operation, and that increased significantly up to 51 (31.7%) at the final follow-up (*P* = 0.04). In the consistency group, the incidence of CM at the final follow-up was much higher than that preoperatively (35:16, *P* = 0.002). CM-free survival time decreased significantly in patients with larger CBD correction, pelvic fixation and more instrumented segments, respectively, during follow-up (*P* < 0.05, log-rank test). Age ≥ 60 years, the consistency CA, pelvic fixation, CBD-correction ≥ 30 mm and fixed-vertebra ≥ 8 were risk factors for CM happening after surgery using multivariate regression analysis (*P* < 0.05).

**Conclusions:**

The coronal alignments in ASD patients underwent long-fusion surgeries may deteriorate during follow-up, for which the risk factors include the consistency CA, age ≥ 60, fixed-vertebra ≥ 8, CBD-correction ≥ 30 mm and pelvic fixation.

## Introduction

The prevalence of coronal malalignment (CM) is very common, ranging from 19% to 40% [1, 2], which probably undermine the quality of life (QoL) significantly in patients with adult spinal deformity (ASD) before and after surgery [3–6]. Achieving coronal balance in patients underwent the surgical procedure of long-fusion with instrumentations would be difficult unless the risk factors for the postoperative CM were recognized [1, 2, 7]. Those surgical risk factors including the correction in major curve and the ability to level the L4 and L5 coronal tilt have been reported recently [2, 8]. Unfortunately, there was a paucity of study aiming to investigate whether the coronal alignments (CA) postoperatively would be deteriorating during follow-up?

In this current study, we investigated a series of ASD patients underwent the surgical procedure of long-fusion with instrumentations to explore whether the CA postoperatively will be deteriorating? And to identify the risk factors for CM-developing during follow-up.

## Materials and methods

Approvals by the Clinical Research Ethics Committee of our hospitals was obtained before this research. We retrospectively reviewed the data of ASD patients treated in the three institutions from March 2019 to May 2020. All of those participants concerned in this current study had undergone the surgical procedure of long-fusion (≥ 5 vertebras) with instrumentations via posterior approach only.

General inclusion criteria for this study were as follows: Patients suffering from ASD with (1) age ≥ 40 years; (2) integrated data; and (3) follow-up duration ≥ 24 months after surgery. Those individuals had spinal surgeries previously, spinal neoplasms, spinal tuberculosis, spinal or pelvic trauma, non-structural curvature, prior hip or knee surgeries or the discrepancy between lower extremities ≥ 2 cm were excluded from this study.

Demographic data including age, gender, body mass index (BMI) and comorbidities were documented. Surgical data included the upper instrumented vertebra (UIV), lower instrumented vertebra (LIV), the grade of osteotomy and the number of instrumented vertebras. Moreover, the follow-up duration and the CM-free survival time after surgery were recorded as well.

### Surgical procedures

All cases in this study had primary coronal deformities and were performed the correction surgery using posterior pedicle screws and 2-rod constructs (titanium alloy). Pedicle screws were placed at all levels in the construct. Pelvic fixation was achieved with either S1 screws, iliac wing screws or S2-iliac screws. Pedicle subtraction osteotomy [9] and vertebral column decancellation [10] were performed in the apex of the major curve in the deserved patients, respectively. The surgical procedures of posterior lumbar interbody fusion (PLIF) or transforaminal lumbar interbody fusion (TLIF) were used at the lower lumbar spine on the concavity if it was deemed necessary to augment fusion as an anterior column support, or if foraminal compression was present, whereby the cage was used to increase the foraminal area.

### Radiographic data

Radiographic data, including full-length coronal and sagittal radiographs, were obtained in free-standing posture with the upper limbs resting on a support, the shoulders at 30° forward flexion, and the elbows slightly flexed [11]. All of the radiographic parameters were measured with the Surgimap Software (version: 2.3.2.1; Spine Software, New York, NY).

**Fig. 1:**
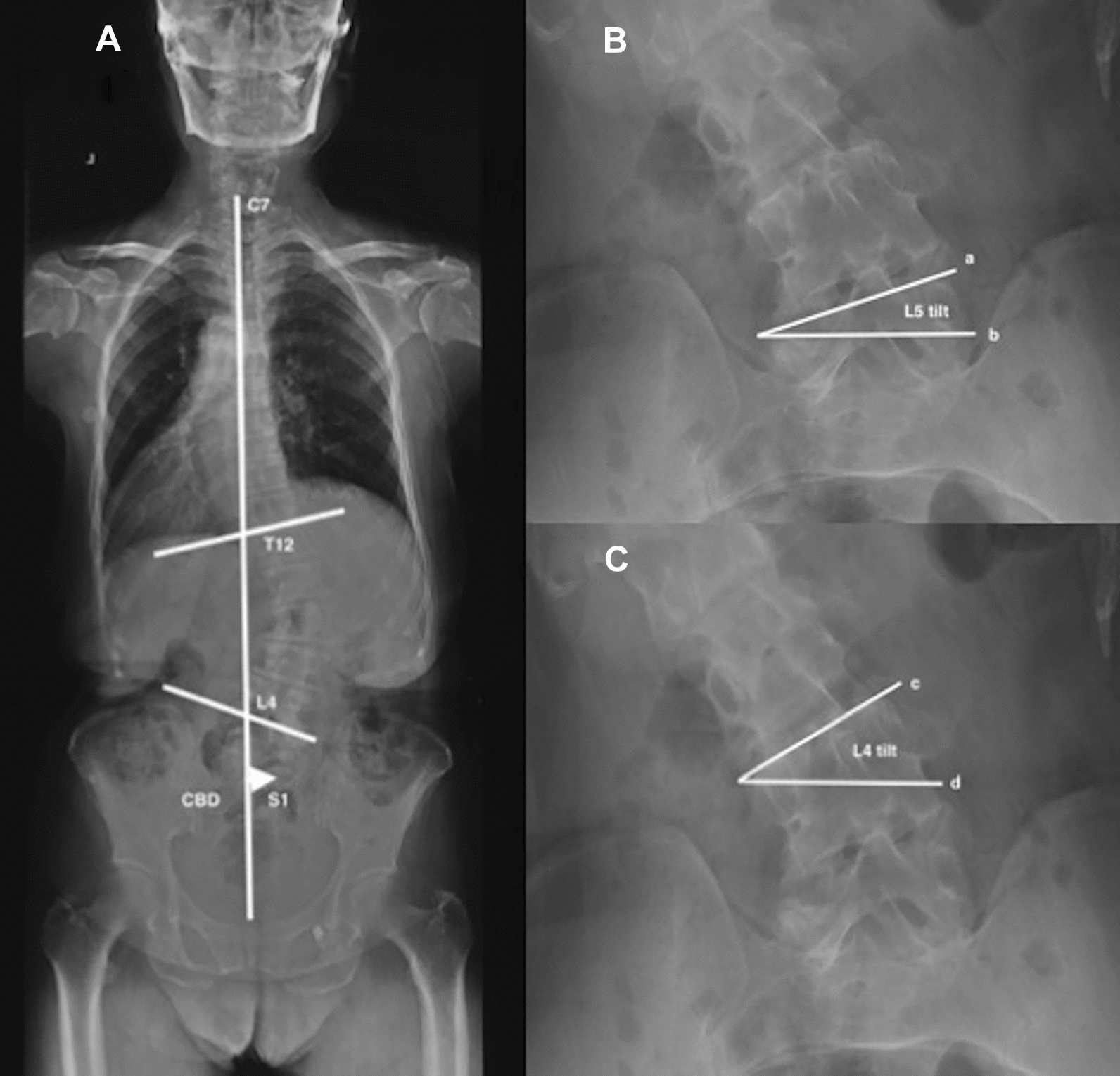
Schematic drawing of each coronal parameter. Coronal balance distance (CBD), the major Cobb angle (**A**), L4 coronal tilt (**C**), and L5 coronal tilt (**B**)

4 coronal parameters were recorded as follows:

1. Major Cobb (MC), the Cobb angle between the superior endplate of the most tilted vertebra cranially and the inferior endplate of the most tilted vertebra caudally (left scoliosis was recorded as negative [−], right scoliosis as positive [+]).
2. Coronal balance distance (CBD), the horizontal distance between the middle of S1 and the C7 plumb line (C7PL). If the C7PL locating at the left of S1, CBD was recorded as the negative value (−), or else, as the positive value (+).
3. L4 coronal tilt, the angle between superior endplate of L4 and the horizontal line (left side was recorded as the negative value [−], and right side as the positive value [+]).
4. L5 coronal tilt, the angle between superior endplate of L5 and the horizontal line (left side was recorded as the negative value [−], and right side as the positive value [+]). Figure 1A–C

The correction of those parameters were calculated by the formula shown as follows:

$${\text{d}} - {\text{variable}} = {\text{post}} - {\text{variable}}{-}{\text{pre}} - {\text{variable}}$$

### Statistical analysis

**Fig. 2:**
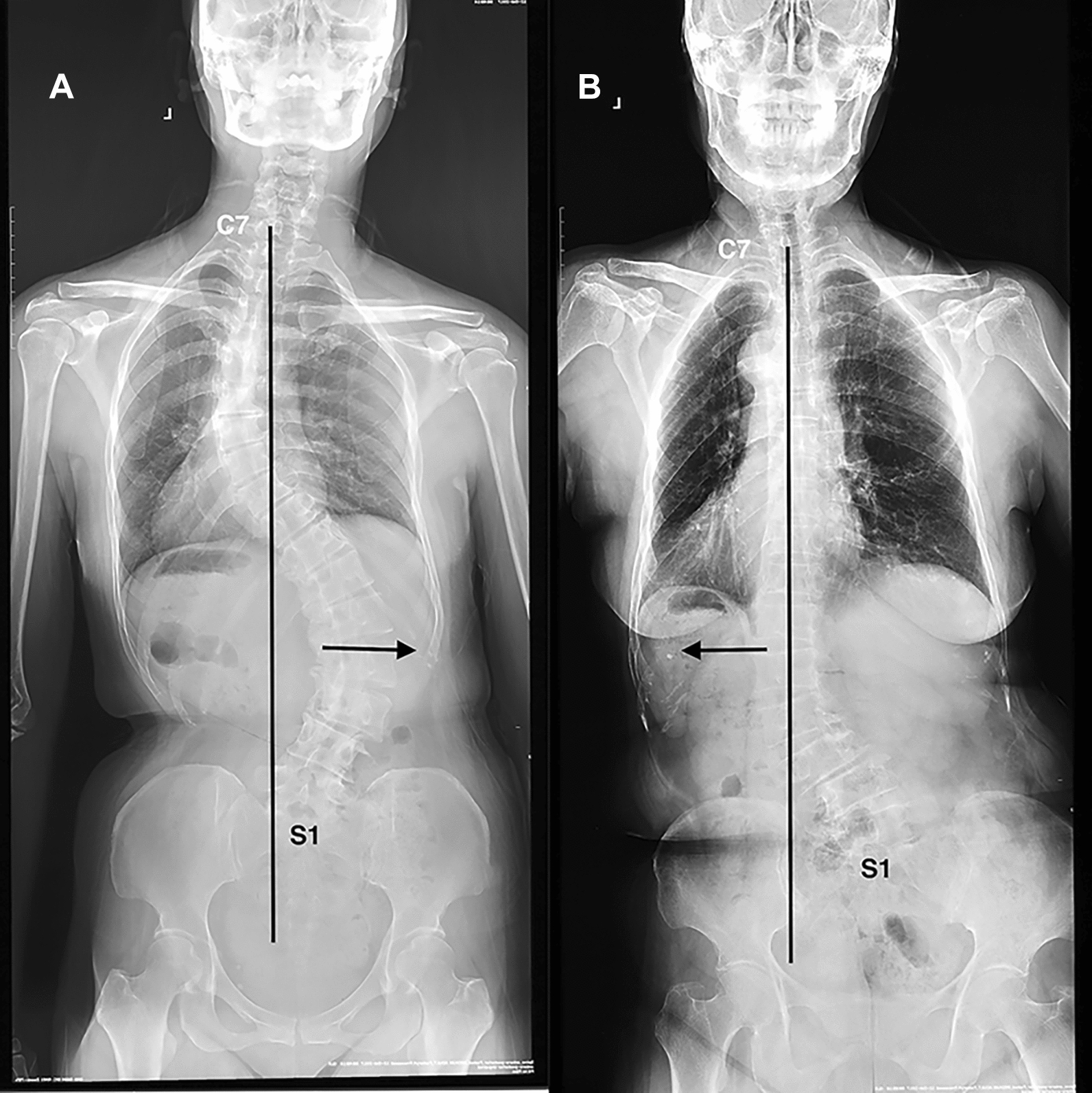
Coronal patterns based on the relationship between orientation of major curve and C7 plumb line. Opposition coronal alignment (**A**) and Consistency coronal alignment (**B**)

According to the orientation of major curve being relative to the C7 plumb line (C7PL) shifting on coronal images preoperatively, patients were subdivided into the consistency group (C7PL shifting towards the convex side) and the opposition group (C7PL shifting towards the concave side) (Fig. 2A, B) All of those radiographic parameters were measured by two independent spinal surgeons from the surgical team. Intra- and inter-rater reliabilities were excellent with the kappa values ranging from 0.889 to 0.938.

According to the results reported in previous studies [1, 2, 12], CBD > 30 mm or < − 30 mm was defined as CM.

Those continuous variables with normal distribution were documented as mean ± standard deviation (SD), or else as the median. Categorical variables were expressed as percentages. Comparisons were performed between the two groups. Chi-square analysis or Fisher’s exact tests were used to assess the differences in categorical variables between the two groups. A Kaplan–Meier curve and log-rank test were used to analyze the differences in CM-free survival during follow-up. Multivariate analysis via a Cox proportional hazards model was used to explore the risk factors for CM-developing. All of the statistical analyses were performed with SPSS 26.0 package software (version for Mac IBM© SPSS^®^ Statistics) with the statistical significance being set at *P* < 0.05.

## Results

A total of 161 patients (45 males, 116 females) with adult spinal deformity (ASD) were included in this study, having the mean age of 64.48 years (SD 8.88, ranging from 40 to 79 years) at surgery. There were 75 cases (46.6%) and 86 cases (53.4%) in the consistency and the opposition group, respectively. Comparisons of those demographics regarding to age, gender and BMI showed no differences between the two groups (Table 1).

**Table 1:** Demographic data in two groups

Variable	Consistency (*n* = 75)	Opposition (*n* = 86)	*P* value
Gender (male: female)	19:56	26:60	0.304
Age at surgery, years	64.1 ± 8.63	64.82 ± 8.46	0.594
Diagnosis, *n*			
Degenerative scoliosis	44	53	0.580
Idiopathic scoliosis	18	23	
Other	13	10	
BMI, kg/m^2^	26.1 ± 2.2	26.31 ± 3.1	0.626
Follow-up, months	50.18 ± 15.56	53.2 ± 15.98	0.228
Comorbidities, *n*			
Yes	57	58	0.153
No	18	28	

Value were presented as the mean ± SD; BMI indicates body mass index

### Coronal parameters and surgical factors

There were no differences in those coronal parameters regarding to the MC and CBD preoperatively, and the surgical data such as the osteotomy, instrumented segments, UIV and LIV between the consistency and the opposition group (*P* > 0.05). Postoperatively, the CBD in the consistency group was much larger than that in the opposition group at the immediate post-operation (*P* = 0.013) and the final follow-up (*P* = 0.007), respectively. Moreover, the perioperative correction of MC and CBD in the consistency group was 17.39° (SD 10.24°) and 23.41 mm (SD 19.46 mm) compared with that of 13.84° (SD 8.30°) (*P* = 0.016) and 17.15 mm (SD 16.20 mm) (*P* = 0.027), respectively.

L4 coronal tilt in the consistency group was much larger than that in the opposition group at the pre-operation (*P* < 0.001), post-operation (*P* = 0.008) and the final follow-up (*P* = 0.006), so was the correction in L4 tilt (*P* = 0.004). L5 coronal tilt preoperatively and its correction were 8.99° (SD 5.97°) and 4.58° (SD 4.31°) in the consistency group compared with that of 7.14° (SD 4.53°) and 3.29° (SD 2.95°) in the opposition group (*P* = 0.027, 0.026), respectively.

### CM developing and those risk factors

The number of patients showing CM at pre-operation was 35 (21.7%), and that increased up to 51 (31.7%) (*P* = 0.04) at the final follow-up. The incidence of CM in the consistency group was much higher than that in the opposition group at the immediate post-operation (*P* = 0.037) and the final follow-up (*P* < 0.001). Comparisons of the incidence of CM in the consistency group between the pre-operation and the final follow-up had significant differences (*P* = 0.002), which had no difference in the opposition group although. Those details are listed in Table 2.

**Table 2:** Surgical features in the two group

Variables	Consistency group (*n* = 75)	Opposition group (*n* = 86)	*P*
Coronal malalignment, *n*			
Pre-operation	16	19	0.907
Post-operation	27	18	0.037*
Final follow-up	35	16	< 0.001*
			0.002*^Δ^; 0.705^♦^ 0.04*^Ψ^
CBD, mm			
Pre-operation	16.13 ± 15.37	17.35 ± 16.62	0.631
Post-operation	24.20 ± 18.33	18.23 ± 11.65	0.013*
Final follow-up	26.89 ± 21.20	19.67 ± 12.23	0.007*
CBD correction	23.41 ± 19.46	17.15 ± 16.20	0.027*
Major Cobb, °			
Pre-operation	26.19 ± 12.78	25.57 ± 12.62	0.758
Post-operation	10.56 ± 7.33	11.69 ± 8.24	0.362
Final follow-up	10.98 ± 7.12	11.78 ± 8.31	0.516
Major Cobb correction	17.39 ± 10.24	13.84 ± 8.30	0.016*
L4 coronal tilt, °			
Pre-operation	16.07 ± 7.83	12.01 ± 6.86	< 0.001*
Post-operation	8.81 ± 4.73	6.83 ± 4.64	0.008*
Final follow-up	8.75 ± 4.96	6.74 ± 4.16	0.006*
L4 tilt correction	8.49 ± 6.42	6.11 ± 3.9	0.004*
L5 coronal tilt, °			
Pre-operation	8.99 ± 5.97	7.14 ± 4.53	0.027*
Post-operation	5.75 ± 3.96	4.74 ± 4.16	0.118
Final follow-up	5.86 ± 3.88	4.67 ± 4.52	0.077
L5 tilt correction	4.58 ± 4.31	3.29 ± 2.95	0.026*
Osteotomy segments	2.41 ± 1.18	2.16 ± 0.94	0.137
Instrumented segments	8.04 ± 2.3	8.39 ± 2.0	0.303
UIV, *n*			
T10 or above	45	48	0.633
T11–L1	30	38	
LIV, *n*			
L5	20	32	0.178
Pelvic fixation	55	54	

All of the parameters are numeric value. * indicates *P* < 0.05; ^Ψ^comparison of all patients with CM between the pre-operation and the final follow-up; ^Δ^comparisons between the pre-operation and the final follow-up in the consistency group; ^♦^comparisons between the pre-operation and the final follow-up in the opposition group; CBD, coronal balance distance; UIV, upper instrumented vertebra; LIV, lower instrumented vertebra

**Fig. 3:**
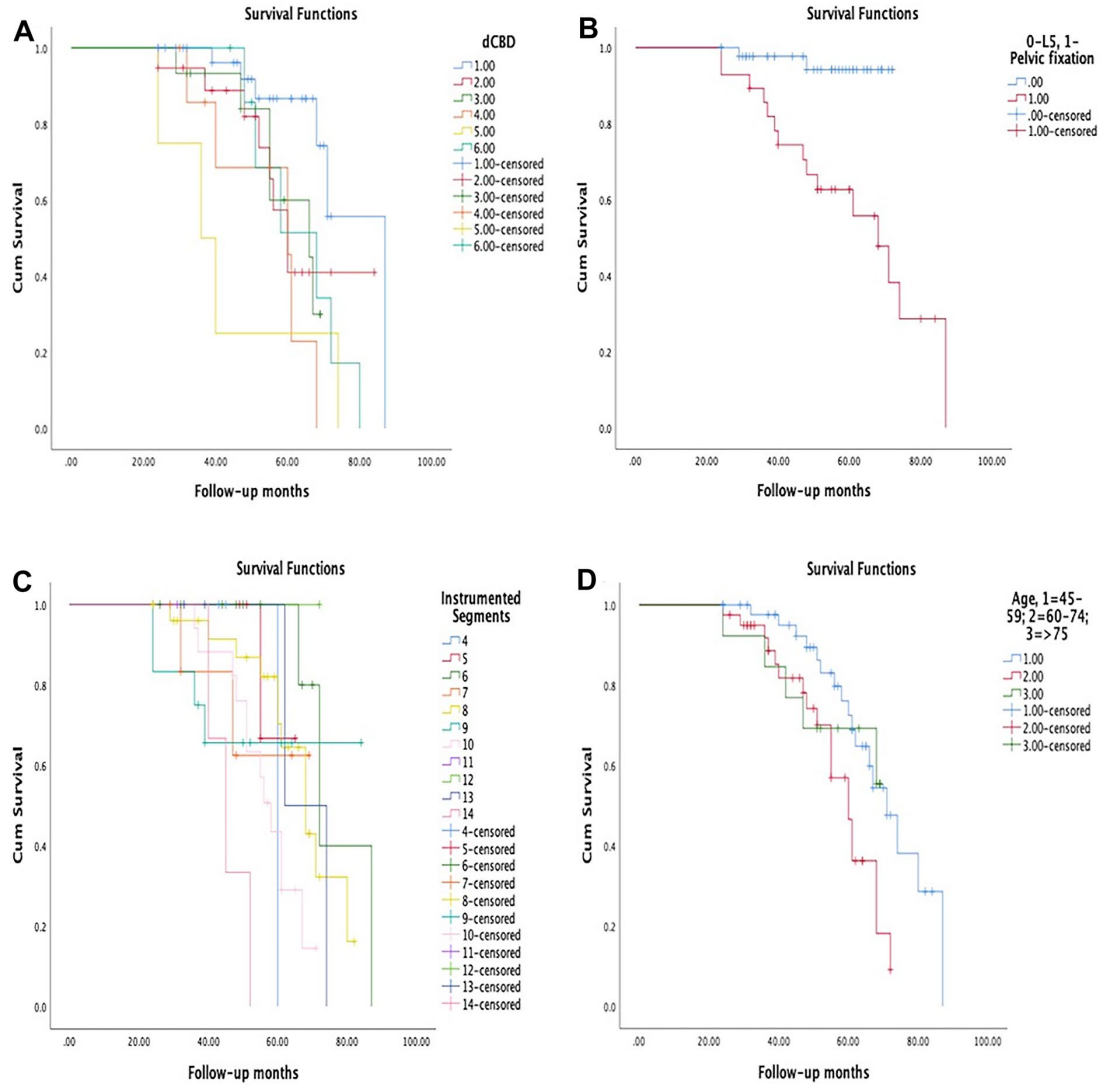
**A** Kaplan–Meier curves of time periods without coronal malalignment (CM) stratified all patients by different CBD correction (1, 1–10 mm; 2, 11–20 mm; 3, 21–30 mm; 4, 31–40 mm; 5, 41–55 mm; 6, > 55 mm). **B** Kaplan–Meier curves of time periods without CM stratified all patients by location of the LIV (L5 or pelvic fixation). **C** Kaplan–Meier curves of time periods without CM stratified all patients by instrumented segments. **D** Kaplan–Meier curves of time periods without CM stratified all patients by age (1, 45–59 years; 2, 60–74 years; 3, ≥ 75 years)

CM-free survival time decreased significantly in patients with increasing correction of CBD (*P* = 0.023, log-rank test) (Fig. 3A), pelvic fixation (*P* = 0.027, log-rank test) (Fig. 3B) and increasing fusion segments (*P* = 0.017, log-rank test) (Fig. 3C) during follow-up. Moreover, stratifying all patients by each of 15 years, the CM-free survival time decreased significantly with aging (*P* = 0.028, log-rank test) (Fig. 3D). Furthermore, after entering the variables into multivariate analysis via a Cox proportional hazards model, the consistency CA (HR 3.896, 95% CI 1.317–11.53), age ≥ 60 years (HR 1.547, 95% CI 1.006–2.381), pelvic fixation (HR 2.824, 95% CI 1.215–6.563), CBD correction ≥ 30 mm (HR 1.364, 95% CI 1.117–1.667) and instrumented segments ≥ 8 (HR 1.069, 95% CI 0.715–1.599) were the risk factors for CM developing (Table 3).

**Table 3:** Multivariate analysis via a Cox proportional hazards model

Variable	HR	95% CI	*P* Value
Age ≥ 60 years	1.547	1.006–2.381	0.04^*^
Consistency alignment	3.896	1.317–11.53	0.014^*^
Pelvic fixation	2.824	1.215–6.563	0.016^*^
UIV	0.501	0.098–2.565	0.406
d-CBD ≥ 30 mm	1.364	1.117–1.667	0.002^*^
Instrumented segments ≥ 8	1.069	0.715–1.599	0.008^*^
L4 coronal tilt	0.911	0.798–1.04	0.168
d-L4 tilt	1.059	0.936–1.199	0.363
L5 coronal tilt	1.164	0.973–1.394	0.097
d-L5 tilt	1.073	0.869–1.326	0.512
d-Cobb	0.968	0.911–1.029	0.299

*Indicates *P* < 0.05; UIV, upper instrumented vertebra; CBD, coronal balance distance; d-, the changes of variables perioperatively

**Fig. 4:**
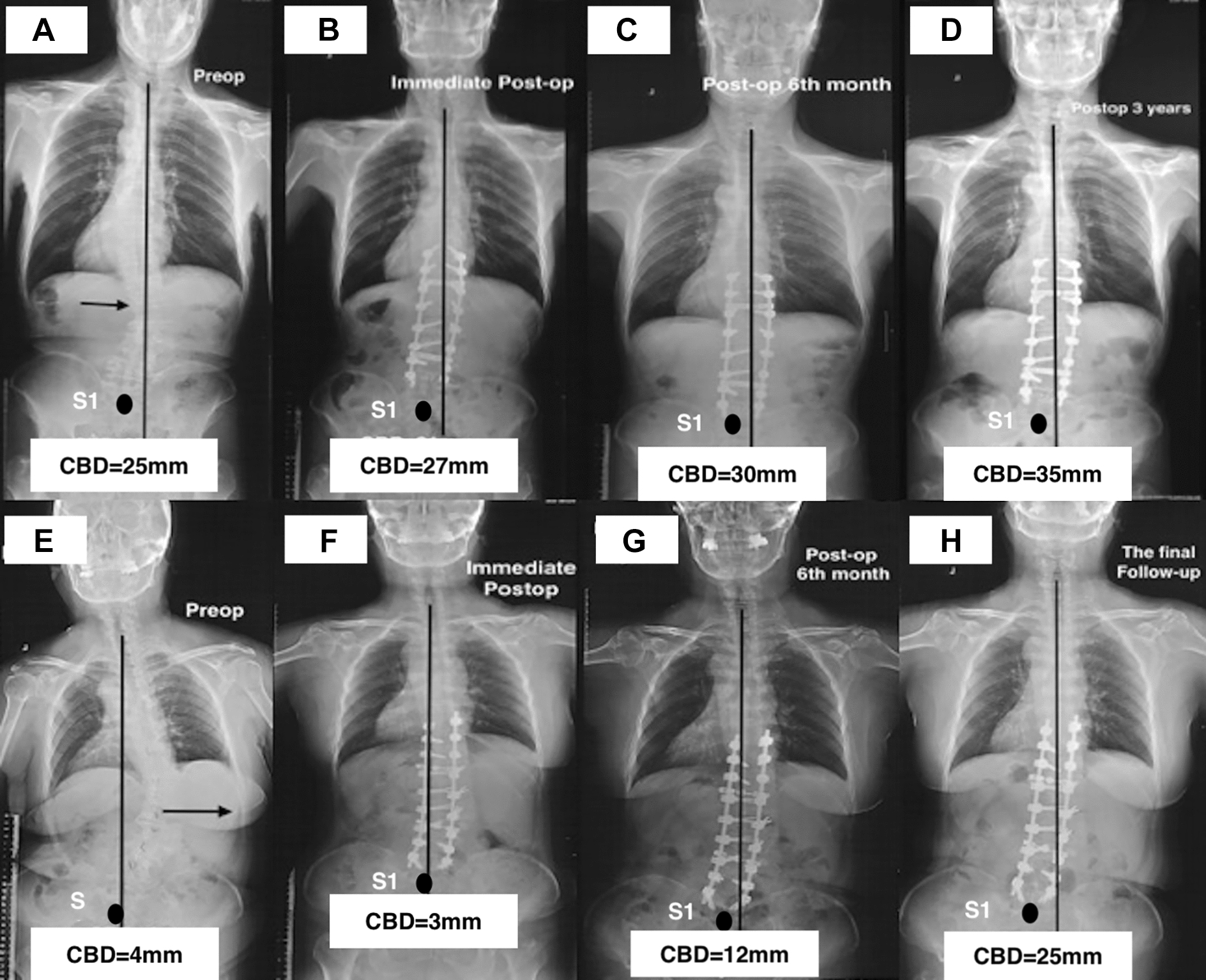
A 61-year-old female ASD patient with consistency alignment had a CBD of 25 mm pre-operatively (**A**), 31 mm immediately (**B**), 30 mm (**C**) and 35 mm (**D**) at 6th month and the final follow-up postoperatively. A 68-year-old female ASD patient with opposition coronal alignment had a CBD of 4 mm preoperatively (**E**), 3 mm immediately (**F**), 12 mm (**G**) and 25 mm (**H**) at 6th month and the final follow-up postoperatively

**Fig. 5:**
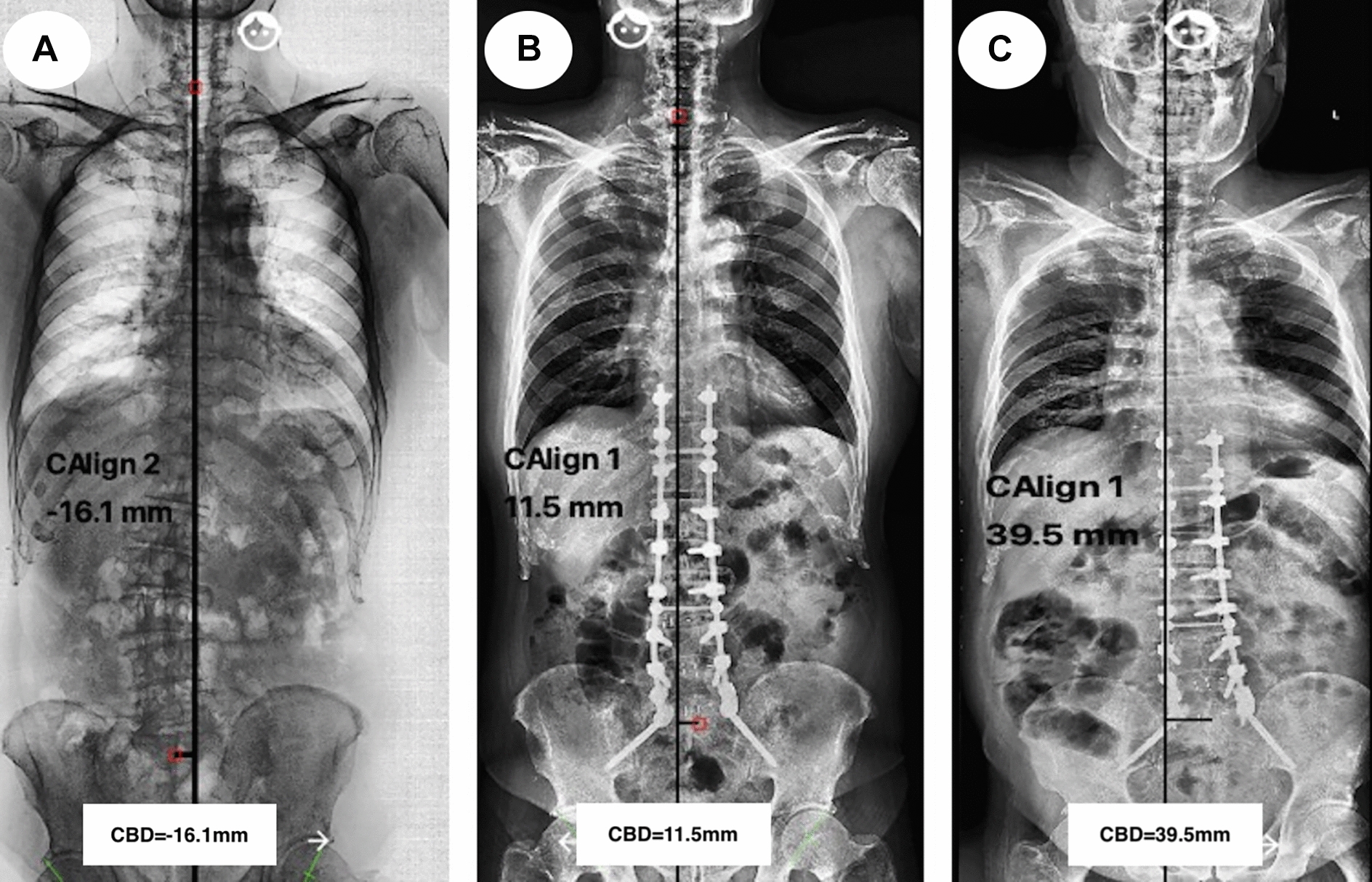
A 68-year-old male ASD patient underwent long-fusion correction surgery (T10–S2), the CBD was − 16.1 mm at pre-operation (**A**), changed to 15.5 mm at the immediate post-operation (**B**), and increased to 39.5 mm at the final follow-up (**C**)

Two representative patients are shown in Figs. 4A–H and 5A–C.

## Discussion

More recently coronal malalignments (CM) in adult spinal deformity (ASD) have been reported, with new classification systems developed to further understand the effects of coronal deformities on pain, physical function and quality of life (QoL) [1, 7, 13]. The surgical procedure of long-fusion with instrumentations can restore the full-body alignments effectively in ASD [3, 14, 15]. Previous studies suggested that those individuals with the consistency coronal alignments (CA) preoperatively may be at the greatest risk for the immediate CM-happening postoperatively after scoliosis surgeries [1, 7]. Therefore, we divided all subjects into the consistency group and the opposition group in this current study. Comparisons between the two groups showed that the CBD at the pre-operation had no differences; however, the CBD of the consistency group at the immediate post-operation and the final follow-up were much larger. Moreover, patients showing CM at the pre-, and post-operation were 35 (16 cases in the consistency group) and 45 (27 cases in the consistency group), respectively, and that increased up to 51 cases (35 cases in the consistency group) at the final follow-up. Comparing the incidence of CM preoperatively and that at the final follow-up, there were significant increases both in all cases and in the consistency group. Furthermore, after multivariate analyzing, the consistency CA was identified as the risk factor for CM-developing during follow-up. As shown in Fig. 4, a 61-year-old female ASD patient with the consistency CA had coronal balance both at the pre-operation and the immediate post-operation (Fig. 4A and B). During the follow-up, the CA gradually deteriorated, the CBD increasing up to 30 mm (Fig. 4C) at the 6th month after the surgery, and CM appeared at the final follow-up (CBD = 35 mm) (Fig. 4D). However, a 68-year-old female ASD patient underwent the similar thoracolumbar fusion surgery, maintained a benign CA throughout the follow-up (Fig. 4E–H). Accordingly, we insist that the consistency CA would be the risk factor for CM-developing in ASD underwent the surgical procedure of long-fusion with instrumentations.

Lewis et al. [8] revealed that the ability to level the L4 and L5 tilt had the greatest impact on the restoration of coronal balance postoperatively in ASD patients underwent long-fusion surgeries. In this current study, the L4 tilt of the consistency group were much larger than that in the opposition group at the pre-, post-operation and the final follow-up, so was the correction in L4 and L5 tilt perioperatively. The incidence of CM in the consistency group was much higher at the pre-, post-operation and the final follow-up. Accordingly, the less correction in L4 and L5 tilt would induce the immediate CM happening after correction surgery. Interestingly, the L4 and L5 tilt and their corrections perioperatively were not the risk factors for CM-developing during follow-up after multivariate analyzing.

Buell et al. [16] suggested that those ASD patients underwent long sacropelvic fusion with the UIV of upper thoracic spine had worse QoL than those with lower thoracic UIV. Furthermore, according to the results reported in previous studies [17–20], lumbosacral and sacroiliac joints were the significant compensatory mechanisms for maintaining the global coronal balance in patients with spinal deformity. In this current study, CM-free survival time in patients with pelvic fixation and more fusion segments (≥ 8) decreased significantly after Kaplan–Meier survival analyzing. As a result, we proposed that those ASD patients underwent long-fusion extending to the pelvis would have less ability to keep the full-body alignments. Because patients suffering from CM have few compensatory mechanism available for this specific condition. The only natural compensatory mechanism is the limited contralateral knee and hip flexion, which are always extremely uncomfortable and cannot be maintain for a long time. In addition, the results illustrated that both the pelvic fixation and more instrumented segments (≥ 8) were the risk factors for CM-happening during follow-up.

Thomapson et al. [21] insisted that the incidence of CM postoperatively could be reduced by avoiding overcorrection in coronal deformity for patients who had undergone long-fusion surgeries with instrumentations. In our current study, CM-free survival time decreased significantly in patients with CBD correction increasing after Kaplan–Meier survival analyzing. Moreover, over correction of CBD (≥ 30 mm) were the risk factors for CM-happening during follow-up according to the multivariate analysis via a Cox proportional hazards model. A male patient with ASD underwent long-fusion surgery (T10–S2) in Fig. 5 showed that CBD was corrected from − 16.1 mm preoperatively (Fig. 5A) to 15.5 mm immediate postoperatively (Fig. 5B), and the CBD increased up to 39.5 mm (Fig. 5C) at the final follow-up. Moreover, the pelvic fixation and instrumented vertebras over 8 in this patient may increase the incidence of CM-developing during follow-up.

Ploumis et al. [22] proposed that the presence of osteoporosis was the significant factor for changes of coronal balance in those ASD patients who had undergone long-fusion surgeries. In this current study, the results showed that the CM-free survival time decreased significantly with aging (*P* = 0.028, log-rank test), after stratifying all patients by each of 15 years, and age ≥ 60 were the risk factors for CM-developing during follow-up postoperatively using the multivariate analysis. As a result of, we deduced that those elderly patients suffered from CM-happening during follow-up may because of the presence of osteoporosis. Unfortunately, the bone mineral density was not included in this current study because of the data missing in one of the medical institution.

Neither L4 and L5 tilt preoperatively nor their corrections were the risk factor for CM developing after the multivariate survival analyzing. Therefore, preoperative L4 and L5 tilt and the correction in major Cobb may just affect the immediate coronal alignment [8], and have less effect on coronal alignment changing during the long-term follow-up postoperatively.

Limitations of this current study should be mentioned. First, health-related questionnaires such as SRS-22, short form-36 and Oswestry disability index were not analyzed in this current study, which were not involved in the purposes of this study although. Second, although this study identified those significant risk factors for coronal malalignment developing during 2-year follow-up postoperatively, which should be investigated by further long-term (≥ 5 years) studies including more larger samples. Finally, this current study concluded those risk factors for CM-developing; however, we did not investigated how to avoided them effectively.

## Conclusions

For ASD patients underwent long-fusion surgeries, the consistency coronal alignment preoperatively may be risk predictor for CM developing at the immediate post-operation, for which the reasons may be the unreasonable correction of L4 and L5 tilt perioperatively. The risk predictors for CM-developing during follow-up postoperatively may include the consistency CA, age ≥ 60 years, pelvic fixation, CBD-correction ≥ 30 mm and instrumented vertebras ≥ 8.

## Data Availability

All data generated during this study are available from the corresponding author on reasonable request. There was no data published previously.

## Code availability

Surgimap Software, version: 2.3.2.1; Spine Software, New York, NY; IBM SPSS statistics software, Mac version 26.0, IBM Corp.
